# Photoelectrocatalytic Activity of ZnO-Modified Hematite Films in the Reaction of Alcohol Degradation

**DOI:** 10.3390/ijms241814046

**Published:** 2023-09-13

**Authors:** Vitali A. Grinberg, Victor V. Emets, Natalia A. Mayorova, Aleksey A. Averin, Andrei A. Shiryaev

**Affiliations:** Frumkin Institute of Physical Chemistry and Electrochemistry, Russian Academy of Sciences, Leninsky Prospekt 31, Building 4, 119071 Moscow, Russia; victoremets@mail.ru (V.V.E.); maynat54@mail.ru (N.A.M.); alx.av@yandex.ru (A.A.A.); a_shiryaev@mail.ru (A.A.S.)

**Keywords:** hematite photoanode, zincoxide, electrochemical deposition, photoelectrocatalytic oxidation, methanol, ethylene glycol, glycerol

## Abstract

Thin-film nanocrystalline hematite electrodes were fabricated by electrochemical deposition and loaded with electrodeposited zinc oxide in various amounts. Under visible light illumination, these electrodes demonstrate high activity in the photoelectrochemical degradation of methanol, ethylene glycol and, in particular, glycerol. Results of intensity-modulated photocurrent spectroscopy show that the photoelectrocatalysis efficiency is explained by the suppression of the electron–hole pair recombination and an increase in the rate of photo-induced charge transfer. Thus, zinc oxide can be considered an effective modifying additive for hematite photoanodes.

## 1. Introduction

Progressive pollution of the environment calls for urgent solutions to ecological problems. New industries, including those aiming at the development of “green” chemistry, make a significant contribution to the deterioration of the environmental situation. For instance, the production of biodiesel has increased significantly in recent decades, leading to the formation of large amounts of glycerol, an important pollutant of the aquatic environment, as a by-product [1,2]. In this regard, glycerol degradation and/or its conversion into a functionalized raw material is a very pressing task. Various value-added products may be obtained from glycerol, including organic acids, dihydroxyacetone, etc. [3,4,5,6,7,8]. However, employed approaches, such as pyrolysis or steam reforming, are generally energy-intensive, and in some cases may cause additional pollution through the formation of toxic by-products. Along with the traditional technologies of conversion and degradation of environmental pollutants, methods of purification using sunlight, namely, photocatalysis and its variant—photoelectrochemical oxidation, have been intensively studied in recent years [9,10,11,12].

Semiconducting oxides may be efficient materials for photoelectrooxidation of organic substrates; titanium dioxide and zinc oxide are especially attractive. Their advantages include low cost, non-toxicity, and corrosion resistance at various pH values of the solution [11]. For example, photoelectrochemical glycerol oxidation accompanied by glyceric acid generation was demonstrated using a cobalt-modified zinc oxide as photoanode [13]. However, both titanium dioxide and zinc oxide are semiconductors with a large band gap (3.2 eV and 3.37 eV, respectively); thus, ultraviolet light, making just ~4% of the solar spectrum (on the Earth’s surface), is required for the photoelectrochemical oxidation of substrates. Therefore, the development of photocatalytic systems active in the visible region of the spectrum is an important problem.

Modification of titanium oxide and zinc oxide with various promoting additives (metals, nonmetals, oxides, etc.) allows shifting the absorption spectrum to the visible region, but only to a relatively small depth. In this regard, semiconducting oxides such as hematite (α-Fe_2_O_3_)with a much smaller band gap (E_g_ = 2.2 eV), absorbing light with wavelengths up to 600 nm, are of interest. This oxide has already attracted considerable attention fromresearchers as a promising photoanode for water oxidation, particularly due to its chemical stability in most aqueous solutions at pH > 3 [14,15]. It can also find application for the photoelectrocatalytic decomposition of organic pollutants [16,17] and for the synthesis of valuable products, for example, in the selective photoelectrooxidation of glycerol [9,18]. The studies of hematite photoanodes are also stimulated by theirabundance and low cost.

The disadvantages of the hematite photoanode include a high level of recombination losses during the oxidation of water and organic substrates, which significantly reduces its photoelectrocatalytic efficiency. However, the losses can be markedly suppressed by surface modification of a hematite electrode with metals or oxides. Previously, we have studied the photoelectrocatalytic properties of a thin-film hematite photoanode obtained by the sol–gel method and modified with titanium, cobalt, and bismuth, in the reaction of methanol degradation from an aqueous solution under illumination with visible light [19]. Subsequently, photoanodes were made from hematite obtained by cathode electrodeposition and modified with a small amount of titanium. Their photoelectrocatalytic properties were studied in the reactions of photoelectrooxidation of methanol, ethylene glycol, and glycerol [20]. It was shown that the increase in the photoelectrooxidation rate in the series H_2_O < CH_3_OH < C_2_H_4_(OH)_2_ < C_3_H_5_(OH)_3_ can be explained by the influence of the chemical nature of the depolarizer on both the rate constant and the recombination processes on the surface states of the titanium-promoted hematite photoanode.

In addition to titanium dioxide, zinc oxide can also act as an effective promoting additive for hematite photoanodes. For instance, an increase in the photocurrents upon deposition of ZnO quantum dots on the α-Fe_2_O_3_ surface was noted [21]. The modification of hematite films with zinc oxide led to a significant increase in its conductivity and photoelectrochemical characteristics during water oxidation [22]. However, the photoelectrocatalytic activity of the modified hematite in aqueous solutions shows wide variations between different studies. In addition, the photoconversion efficiency of hematite thin films is still insufficient due to the short carrier diffusion length, low absorption coefficient, high electron–hole recombination rate, and the position of the valence band potential, which is positive with respect to the H^+^/H_2_ reaction.

Although numerous works address hematite doping aimed at the improvement of photoelectrochemical characteristics, they mainly refer to the photoelectrochemical decomposition of water [23,24,25,26,27,28,29,30,31,32,33,34,35,36,37]. As mentioned above, the promotion of a hematite photoanode with titanium led to a significant catalytic effect in the photoelectrooxidation of alcohols of various structures; the largest effect was observed for glycerol [20].The use of other promoters may, eventually, open possibilities for the efficient oxidation of target compounds. At present, no systematic study of various hematite promoters on the oxidation of different alcohols is available. In addition, there are no indications in the literature of the manifestation of correlations of photoelectrocatalysis in the oxidation of water and organic compounds on promoted hematite photoanodes.

Therefore, the study of the efficiency of ZnO-modified hematite photoanodes in photoelectrocatalytic oxidation of organic substrates is of interest. In the present work, we investigated the activity of ZnO-modified hematite films in the reaction of photoelectrocatalytic degradation of methanol, ethylene glycol, and glycerol under visible light illumination. The photoanode samples were fabricated by electrochemical deposition of hematite films onto the glass substrate with an electrically conductive coating of fluorine-stabilized tin dioxide (FTO glass), followed by loading the hematite film with different amounts of zinc oxide. To study the photoelectrocatalytic activity of ZnO-modified hematite films, both stationary and non-stationary photoelectrochemical methods were used. Using, in particular, the intensity modulated photocurrent spectroscopy (IMPS), the efficiency of recombination loss suppression in reactions of photoelectrocatalytic oxidation of water was quantitatively studied as a function of the amount of the zinc oxide promoter. The influence of the chemical nature of the depolarizer on the recombination losses was studied in reactions involving water and alcohols of different structures. For the studied systems, the corresponding rate constants for charge transfer and recombination were determined. The stability of a zinc-oxide-promoted hematite photoanode in the reaction of photoelectrocatalytic oxidation of glycerol has been shown.

Note that the current study does not address the composition of the products of photoelectrooxidation of the mentioned alcohols.

## 2. Results and Discussion

In general, the preparation of film photoanodes by electrochemical deposition has a number of undeniable advantages, since it makes possible deposition of films at fixed parameters (electrolyte composition, potential of the conductive substrate, temperature, deposition time, etc.). This favors the reproducible production of films with a desired thickness, chemical composition, as well as structure and morphology. However, despite these advantages, it is difficult to predict the photoelectrocatalytic properties of the resulting photoanodes due to the propensity of recombination of photogenerated charges both in the sample’s bulk and surface. In addition, the chemical nature of the oxidized organic substrate also plays a role in the photoelectrocatalysis of its degradation [20]. It is also impossible to predict a priori the optimal amount of the modifying additive, in this case, zinc oxide, on the hematite surface to ensure the maximum photoelectrocatalytic activity of the photoanode. Therefore, to study the activity of a hematite photoanode modified with zinc oxide, it was necessary to test photoanodes with the amount of deposited ZnO in a fairly wide range.

Several photoanode samples were fabricated for the study. The technique used is described in detail in Section 3. Samples with electrodeposited films of pure hematite and pure zinc oxide are designated below as Fe_2_O_3_/FTO and ZnO/FTO, respectively. Samples of photoanodes with films of modified hematite are denoted as ZnO(0.07)/Fe_2_O_3_/FTO, ZnO(0.2)/Fe_2_O_3_/FTO and ZnO(0.87)/Fe_2_O_3_/FTO where the number in parentheses denotes the amount of electricity spent on the electrodeposition of ZnO in coulombs per cm^2^ of the geometric surface of the photoanode. The mole % of zinc in the prepared ZnO-modified photoanodes was estimated; the obtained values are shown in Table 1 below.

### 2.1. Characterisation of the Samples

**Figure 1:**
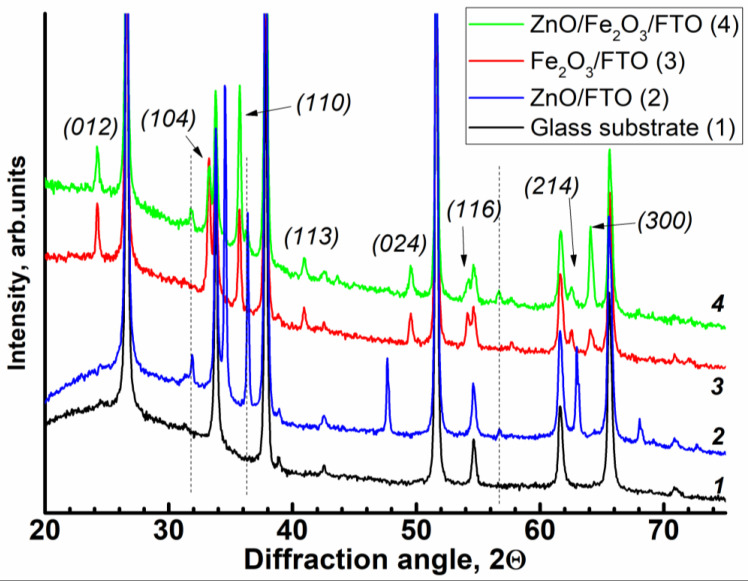
X-ray diffraction patterns of the samples: (*1*) FTO glass substrate, (*2*) ZnO/FTO, (*3*) Fe_2_O_3_/FTO, and (*4*) ZnO(0.2)/Fe_2_O_3_/FTO. Thin dashed vertical lines denote main peaks of ZnO; arrows show principal reflections of hematite. The curves are displaced vertically for clarity; the intensity axis is zoomed to highlight the contribution of the film.

X-ray diffraction patterns of the conductive FTO glass substrate (*1*) and of the deposited films: Fe_2_O_3_/FTO (*2*), ZnO/FTO (*3*), and ZnO(0.2)/Fe_2_O_3_/FTO (*4*) are shown in Figure 1. The glass pattern is dominated by SnO_2_. The patterns of the hematite films modified with different amounts of ZnO (0.07, 0.2 and 0.87 C cm^−2^) contain peaks due to rhombohedral α-Fe_2_O_3_ and ZnO. The hematite peaks observed at 24.1°, 33.1°, 35.6°, 40.9°, 49.4°, 54.0°, 64° (2θ) are assigned to (012), (104), (110), (113), (024), (116), (300) reflections, respectively (see ICDD card № 33-0664; space group 167, lattice parameters: a = b = 5.03, c = 13.74 Å). The (104) and (110) peaks dominate as expected for the “ideal” powder of the oxide. The zinc oxide peaks correspond to the hexagonal phase (space group 186; lattice parameters: a = b = 3.2498, c = 5.206 Å). No traces of other phases, for example, of the ZnFe_2_O_4_ phase, are observed even in measurements with high statistics. The size of zincite crystallites as inferred from the Scherrer formula is close to 20 nm; the hematite crystallites are approximately two times larger. However, the (101) reflections are somewhat broader, which might indicate larger sizes in this crystallographic direction. We note that the interlayer (101) spacing of ZnO is 2.47 Å, which is close to double the (110) hematite spacing. With great caution, one might propose a kind of epitaxial relationship between the hematite and ZnO.

**Figure 2:**
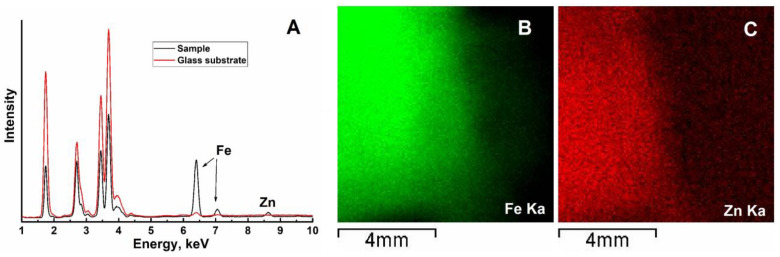
Results of X-ray fluorescence analysis: (**A**) part of the EDX spectrum highlighting the region of Fe and Zn lines; (**B**,**C**) intensity maps of Fe K_α_ and Zn K_α_ lines, respectively.

The presence of Fe and Zn in the deposited film samples was confirmed by X-ray fluorescence analysis (Figure 2A). The maps of Fe Kα and Zn Kα distribution are shown in Figure 2B,C. The distribution of both elements coincides, indicating their successive deposition. Extraction of the Fe/Zn ratio from the present XRF data would be highly unreliable: since the energy of the employed analytical X-ray lines is relatively high (>6 keV), the depth of the analyzed volume exceeds the thickness of the deposited films. In addition, the density of the layer is unknown; furthermore, cracks in the films also hamper the quantitative evaluation of the data.

**Figure 3:**
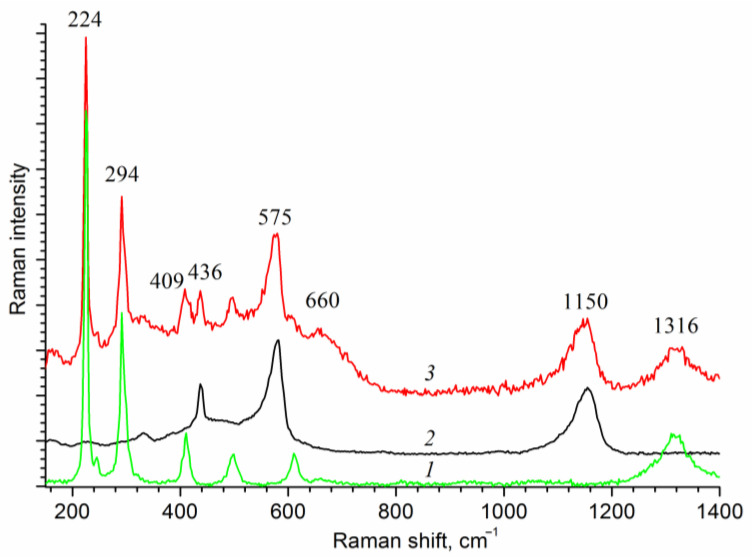
Raman spectra of the film samples: (*1*) Fe_2_O_3_/FTO; (*2*) ZnO/FTO; and (*3*) ZnO(0.2)/Fe_2_O_3_/FTO.

Figure 3 shows the Raman spectra of the deposited films. The observed characteristic peaks at 436 cm^−1^ and 575 cm^−1^ belong to hexagonal zinc oxide. Small shifts of these peaks from the reference positions may arise from substrate-induced strain [38]. Note that the characteristic peaks of zinc oxide in the XRD and Raman spectra are observed for all samples of the hematite film loaded with zinc oxide in the range from 0.07 to 0.87 C cm^−2^.

**Figure 4:**
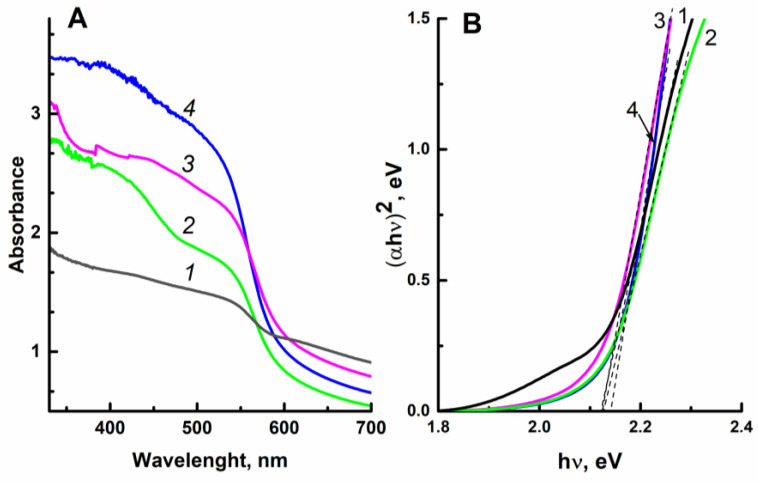
Absorption spectra (**A**) and dependence of (*αhν*)^2^ on photon energy *hν* (**B**) for the film photoanodes: (*1*) Fe_2_O_3_/FTO; (*2*) ZnO(0.07)/Fe_2_O_3_/FTO; (*3*) ZnO(0.2)/Fe_2_O_3_/FTO; and (*4*) ZnO(0.87)/Fe_2_O_3_/FTO.

The absorption spectra of the original and the ZnO-modified hematite films are shown in Figure 4A. To eliminate the effect of the film thickness, the curves shown in Figure 4A were normalized to [0, 1] (see Figure S1). The band gap of the electrodeposited films was estimated in Tauc coordinates [39,40] (see Figure 4B). The direct band gap of a semiconductor (E_g_) can be obtained by extrapolation of the linear part of the function (*αhν*)^2^ to the *x* axis (photon energy *hν*). As seen from Figure 4B, the modification of Fe_2_O_3_ with zinc oxide does not affect the band gap energy; for the studied samples, the E_g_ values are close to 2.12 eV. The intersection of two tangents in determining the band gap of the sample modified with zinc oxide gives a close value of 2.13–2.14 eV. 

The value of the band gap obtained for the ZnO-modified hematite samples is very close to the value of the band gap E_g_ = 2.13 eV for the α-Fe_2_O_3_ calcined at high temperature [36].

**Figure 5:**
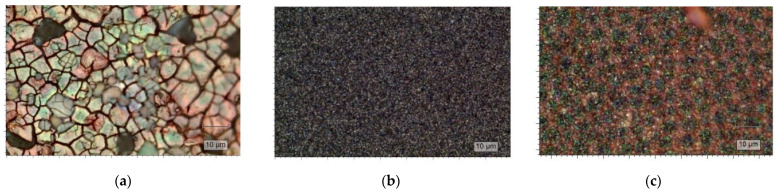
Optical microphotographs of the film samples: (**a**) Fe_2_O_3_/FTO; (**b**) ZnO/FTO; and (**c**) ZnO(0.2)/Fe_2_O_3_/FTO.

Figure 5 shows optical microphotographs of the electrodeposited hematite, zinc oxide and ZnO-modified hematite film. Each film exhibits some surface defects. The structure and morphology of the α-Fe_2_O_3_ and ZnO films, as well as their optical properties, correlate well with the literature data [41,42].

**Figure 6:**
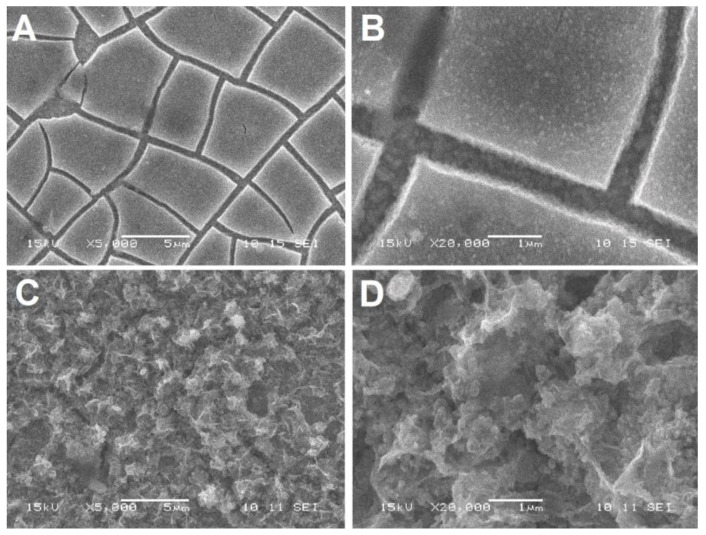
SEM images of the film samples: (**A**,**B**) ZnO(0.2)/Fe_2_O_3_/FTO; and (**C**,**D**) ZnO(0.87)/Fe_2_O_3_/FTO at various resolutions.

The morphology of the deposited films was assessed by Scanning Electron Microscopy (SEM) with a JEOL JSM-6060 SEM (JEOL, Tokyo, Japan). Representative SE (Secondary Electron) images are shown in Figure 6 below and Figure S2 in the Supplementary Materials.

There are noticeable differences between the samples with different content of zinc oxide. The ZnO(0.2)/Fe_2_O_3_/FTO sample (Figure 6A,B) consists of a hematite layer with numerous brittle cracks. The cracks are most likely formed due to the layer contraction after the annealing stage (2 h at 500 °C and 10 min at 750 °C). The hematite layer and the bottom of the crack are “decorated” with tiny (~100 nm and less), mostly isometric, ZnO particles. The sample with a high content of zinc oxide ZnO(0.87)/Fe_2_O_3_/FTO (Figure 6C,D) shows a highly developed extremely uneven surface. With increasing resolution, it can be seen that the surface of the sample ZnO(0.2)/Fe_2_O_3_/FTO consists of approximately identical particles of hematite and zinc oxide (Figure S2A,B). The surface of the ZnO(0.87)/Fe_2_O_3_/FTO sample (Figure S2C,D), even at low resolutions, is completely covered with zinc oxide agglomerates. The SEM image of the ZnO(0.2)/Fe_2_O_3_/FTO sample shows a well-defined morphology with a particle size of approximately 30–50 nm. Note that the morphology of the sample ZnO(0.07)/Fe_2_O_3_/FTO with the smallest amount of electrodeposited zinc oxide is similar to that of the ZnO(0.2)/Fe_2_O_3_/FTO sample. 

Thus, the morphological data obtained show that the sample with the highest zinc oxide content has a surface covered with large particle agglomerates compared to samples with medium and low zinc oxide content. This is reflected in the photoelectrocatalytic activity. The optimal, i.e., medium, zinc oxide content correlates with the minimum particle size (Figure S2).

### 2.2. Influence of the Modifying Component on the Photoelectrocatalytic Oxidation of Water, Methanol, Ethylene Glycol and Glycerol 

When a nanocomposite made of ZnO and α-Fe_2_O_3_ is illuminated, photogenerated electrons migrate from the ZnO conduction band to the conduction band of α-Fe_2_O_3_ [29]; the holes from the α-Fe_2_O_3_ valence band transfer and accumulate in the ZnO valence band. In this case, electrons in the conduction band of α-Fe_2_O_3_ become available for the reduction of protons to hydrogen. Formation of the composite reduces the probability of recombination of the generated charges, thus increasing the performance of the photoelectrochemical cell. Other possible mechanisms of the charge carrier transfer across the ZnO/Fe_2_O_3_ heterojunction are also described in the literature [43,44]. In any case, electronic properties and the structural features (particle size, morphology, presence of defects, etc.) of a composite material are of great importance [45,46].

**Figure 7:**
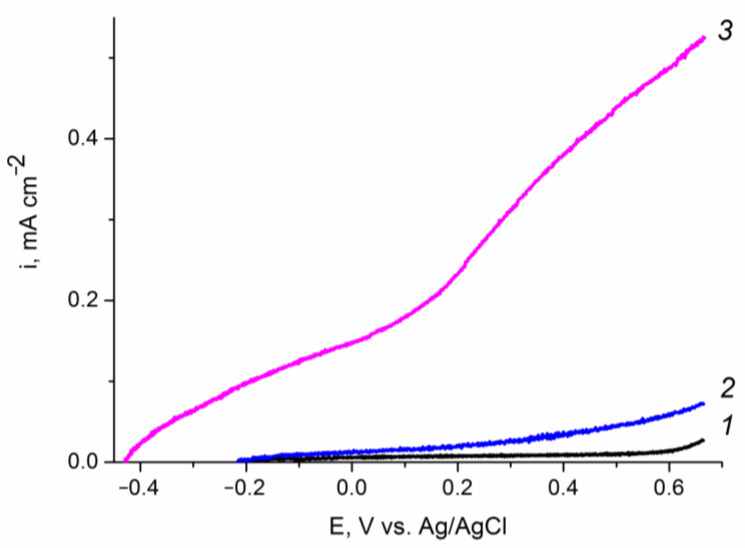
Voltammograms of the (*1*, *2*) Fe_2_O_3_/FTO and (*1*, *3*) ZnO(0.2)/Fe_2_O_3_/FTO film photoanodes in an aqueous solution of 0.1 M KOH obtained: (*1*) in “dark” voltammetry conditions; and (*2*, *3*) under visible light illumination with a power density of 100 mW cm^−2^. Potential scan rate is 10 mV s^−1^. The “dark” curves for the two samples practically coincide.

Preliminary experiments in background electrolyte (Figure 7) showed that the promotion of electrodeposited hematite films with a medium amount of zinc oxide (0.2 C cm^−2^) leads to an increase in the photocurrents of water oxidation at the photoanode by at least an order of magnitude at the potential of 1.23 V (vs. RHE). Modification of hematite with very small (0.07 C cm^−2^) or large (0.87 C cm^−2^) amounts of ZnOalso leads to an increase in water splitting photocurrents, but to a much lesser extent (see Figures S3 and S4 in Supplementary Materials). These results can be explained in terms of an increase in the density of surface states (SS) on a hematite photoelectrode upon its modification with zinc oxide [47]: an increase in the SS density reduces the recombination of photogenerated charges (electrons and holes) and increases the efficiency of charge transfer to water molecules. A similar approach was used in [48], where the SS wasstudied quantitatively and their effect on the efficiency of a cobalt-promotedhematite photoanode was addressed.

**Figure 8:**
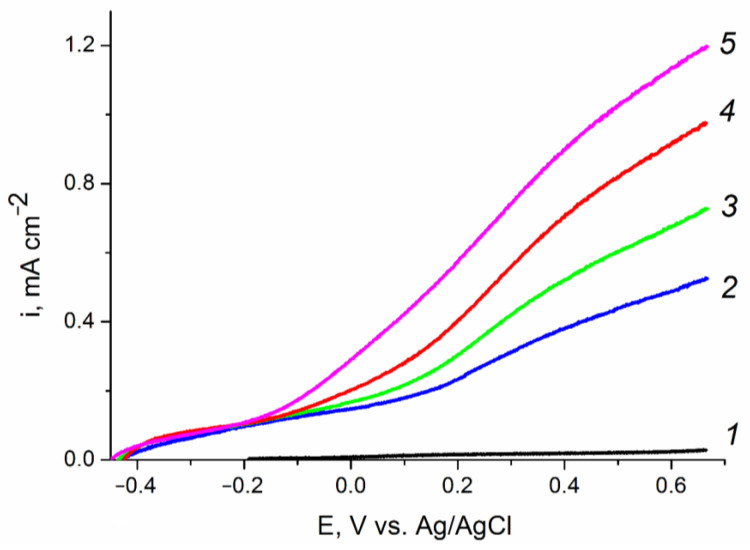
Voltammograms of the ZnO(0.2)/Fe_2_O_3_/FTO film photoanode obtained: (*1*) in «dark» voltammetry conditions in all solutions; and (*2*–*5*) under visible light illumination with a power density of 100 mW cm^−2^ in aqueous solutions of (*2*) 0.1 M KOH;(*3*) 0.1M KOH + 20% CH_3_OH; (*4*) 0.1 M KOH + 20% C_2_H_4_(OH)_2_; and (*5*) 0.1 M KOH + 20% C_3_H_5_(OH)_3_. Potential scan rate is 10 mV s^−1^.

The photoelectrooxidation of other depolarizers, particularly of alcohols with various structures, is also of interest. Figure 8 shows the polarization curves of the photoelectrooxidation of methanol, ethylene glycol, and glycerol from an aqueous solution of 0.1 M KOH on the ZnO(0.2)/Fe_2_O_3_/FTO photoanode sample. For all studied organic substrates, a well-pronounced wave of direct photoelectrochemical oxidation shifted negatively compared to the background curve is observed. The photocurrent at a photoanode potential of 1.23 V (vs. RHE) in solutions containing 20% methanol, 20% ethylene glycol, or 20% glycerol is, respectively, 1.5, 2 or 3 times higher in comparison with that in the 0.1 M KOH solution. The observed increase in the photocurrent indicates acceleration of the photoelectrooxidation reaction in the following sequence: H_2_O < MeOH < C_2_H_2_(OH)_2_< C_3_H_5_(OH)_3_. The results obtained can be explained by the influence of the nature of the depolarizer both on the rate constant of photoelectrooxidation and on the recombination processes on the surface states of the ZnO/Fe_2_O_3_/FTO photoanode.

The current–voltage characteristics of the composite photoanodes with other amounts of the electrodeposited zinc oxide (0.07 and 0.87 C cm^−2^)in the photoelectrocatalytic oxidation reactions of methanol, ethylene glycol, and glycerol are shown in Figures S3 and S4, respectively. As seen from the photovoltammetry data, regardless of the amount of deposited zinc oxide, the photocurrent densities of the substrate photoelectrooxidation increase in the same order: H_2_O < MeOH < C_2_H_2_(OH)_2_ < C_3_H_5_(OH)_3_. However, for all the substrates and in the entire range of photoanode potentials these currents are lower on samples with the smallest (0.07 C cm^−2^) and highest (0.87 C cm^−2^) amounts of zinc oxide. At a low amount of zinc oxide (0.07), the surface state density apparently decreases as compared to that for the ZnO(0.2)/Fe_2_O_3_/FTO sample, which leads to an increase in recombination losses. These lossesprevail on a pure hematite electrode, where currents of the substrates photoelectrooxidation are very small (see Figure S5), andsome increase in the photoelectrooxidation current compared to that in the supporting electrolyte is observed only for ethylene glycol and glycerol, for which the charge transfer rate constants can be higher than the recombination rate constants. At a large amount of zinc oxide (0.87), the SS density also decreasesas compared to the ZnO(0.2)/Fe_2_O_3_/FTO sample, probably due to partial blocking of these surface states by zinc oxide aggregates.

**Figure 9:**
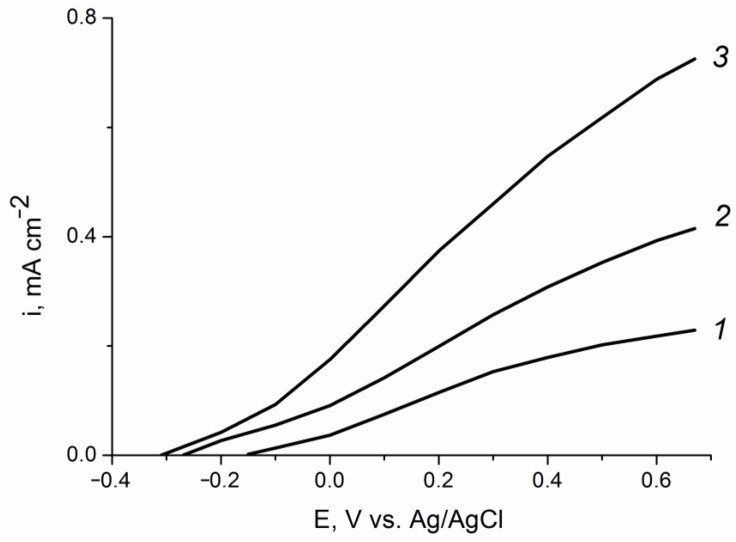
Partial voltammetric curves of the (*1*) ZnO(0.07)/Fe_2_O_3_/FTO, (*2*) ZnO(0.87)/Fe_2_O_3_/FTO, and (*3*) ZnO(0.2)/Fe_2_O_3_/FTO film photoanodes obtained under visible light illumination with a power density of 100 mW cm^−2^ in aqueous solution of 0.1 M KOH + 20% C_3_H_5_(OH)_3_. Potential scan rate is 10 mV s^−1^.

Figure 9 illustrates the influence of the deposited amount of zinc oxide on the photoanode efficiency in the reaction of glycerol photoelectrooxidation. The largest photoelectrocatalytic effect (the most negative shift of the oxidation potential and the highest photocurrent density) is achieved at a composite film obtained upon electrodeposition of ZnO with the consumption of 0.2 C cm^−2^. The oxidation photocurrent density on this sample is 2 and 4 times higher than that on the ZnO(0.87)/Fe_2_O_3_/FTO and ZnO(0.07)/Fe_2_O_3_/FTO samples, respectively. Thus, by selecting the optimal content of the modifying additive, it is possible to significantly increase the activity of the hematite photoanode in the processes of photoelectrooxidation of organic substrates.

In addition, the stability of the ZnO(0.2)/Fe_2_O_3_/FTO photoanode was confirmed in long-term tests. The results of these experiments are illustrated in Figure S6, which shows chronoamperometric curves of the photocurrent of glycerol oxidation at the photoanode potentials 0.4 V and 0.6 V vs. Ag/AgCl. It can be seen from the figure that during the test period(3 h), the photocurrent of glycerol oxidation remains practically constant, which indicates the stability of the photoanode. Furthermore, analysis of the electrolyte solution by Raman spectroscopy in a range of 200–1200 cm^−1^ did not reveal characteristic peaks indicating the presence of iron or zinc ions. 

For the studied ZnO(0.2)/Fe_2_O_3_/FTO photoanode the observed partial photocurrents of glycerol oxidation at a potential of 1.23 V (vs. RHE) in 0.1 M KOH are at least two times higher than those reported for cobalt-promoted zinc oxide and hematite photoanodes (0.74 mA cm^−2^ vs. 0.4 mA cm^−2^ [13] and 0.25 mA cm^−2^ [18], respectively). At the same time, the studied photoanode is still inferior to the BiVO_4_ photoanode, on which the current density of glycerol oxidation of up to 1.0 mA cm^−2^ was obtained in a 0.5 M Na_2_SO_4_ solution at pH 5 and pH 7 [9]. 

Thus, the obtained experimental data show that the highest catalytic activity both in the decomposition of water and in the photoelectrooxidation of various alcohols is demonstrated by a sample of a hematite film with a zinc oxide content corresponding to 0.2 C cm^−2^ electricity spent upon modification. Therefore, this sample was used in subsequent experiments to estimate recombination losses during photoelectrooxidation.

### 2.3. Estimation of Recombination Losses in the Photoelectrooxidation of Alcohols

**Figure 10:**
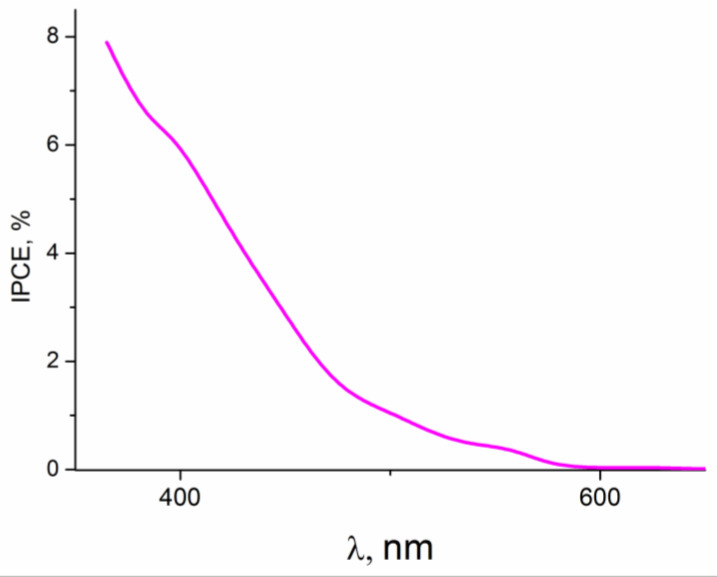
IPCE% spectrum of the ZnO(0.2)/Fe_2_O_3_/FTO photoanode at E = 0.5 V vs. Ag/AgCl in an aqueous solution of 0.1 M KOH + 20% glycerol.

Since the highest photocurrent densities were observed in the photoelectrooxidation of glycerol, the latter was chosen as a model depolarizer for determining recombination losses. The dependence of the quantum efficiency (IPCE%) of the ZnO(0.2)/Fe_2_O_3_/FTO film photoanode on the wavelength of incident monochromatic light obtained in an aqueous solution of 0.1 M KOH + 20% glycerol is shown in Figure 10. As seen from the figure, the sample under study exhibits photoactivity in a wavelength range of 350–600 nm, which is in good agreement with the light absorption data for this sample (Figure 4A, curve *3*). 

To quantify the recombination losses during the photoelectrooxidation of water and alcohols on this photoanode, intensity-modulated photocurrent spectroscopy (IMPS) was used [49,50,51]. When recording the IMPS dependences, monochromatic irradiation with a wavelength of 452 nm (the visible range) was applied, which provided a fairly high value of IPCE% (Figure 10).

**Figure 11:**
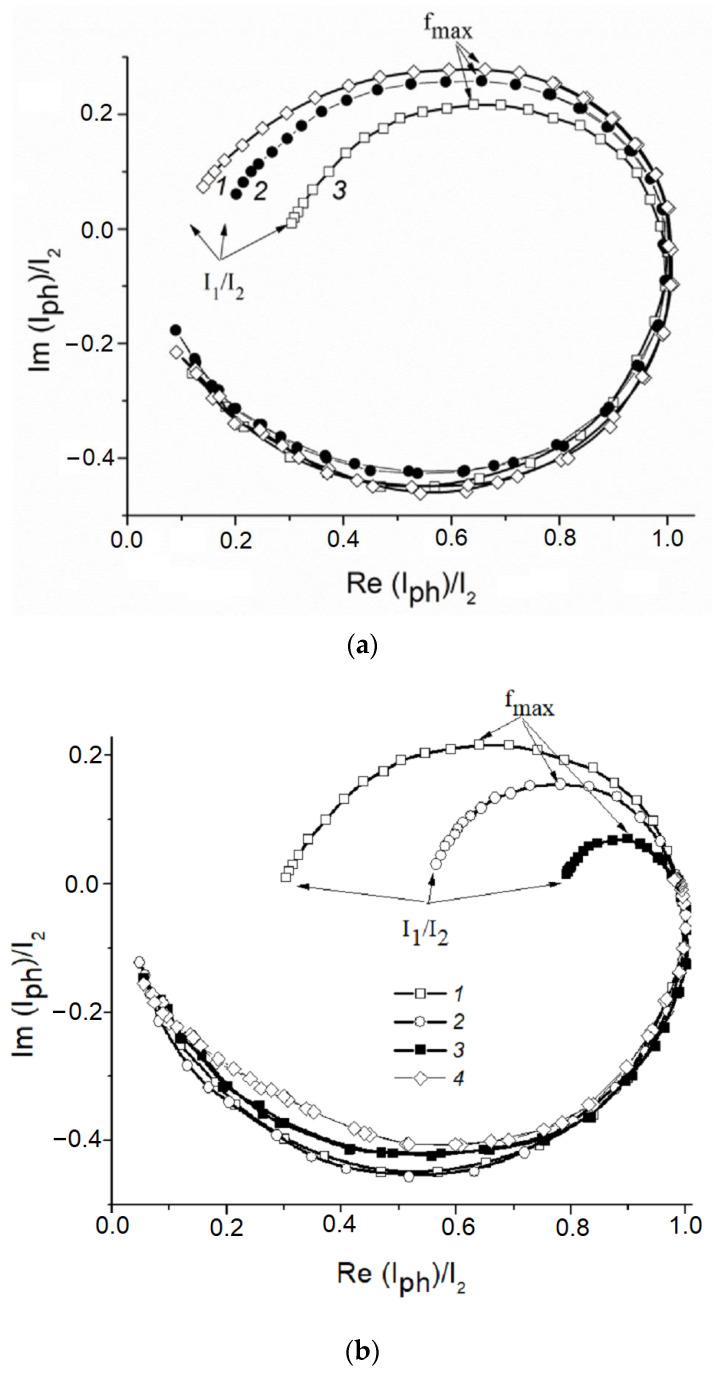
(**a**) Normalized IMPS dependences obtained for the (*1*) ZnO(0.07)/Fe_2_O_3_/FTO; (*2*) ZnO(0.87)/Fe_2_O_3_/FTO; and (*3*) ZnO(0.2)/Fe_2_O_3_/FTO film photoanodes, illuminated with monochromatic light 452 nm at the illumination power density 14 mWcm^−2^ at the photoanode potential 0.5 V in an aqueous solution of 0.1 M KOH. (**b**) Normalized IMPS dependences obtained for the ZnO(0.2)/Fe_2_O_3_/FTO photoanode, illuminated with monochromatic light 452 nm at the illumination power density 14 mW cm^−2^ at a potential of 0.5 V in aqueous solutions of (*1*) 0.1 M KOH; (*2*) 0.1 M KOH + 20%CH_3_OH; (*3*) 0.1 M KOH + 20% C_2_H_4_(OH)_2_;and (*4*) 0.1 M KOH + 20% C_3_H_5_(OH)_3_.

Normalized IMPS curves obtained in the background 0.1 M KOH solution for the ZnO(0.07)/Fe_2_O_3_/FTO, ZnO(0.2)/Fe_2_O_3_/FTO, and ZnO(0.87)/Fe_2_O_3_/FTO photoanode samples indicate significant surface recombination of photogenerated charge carriers during water photoelectrooxidation (Figure 11a).

From the IMPS data, rate constants of the recombination K_rec_ and charge transfer K_ct_ can be calculated. The low-frequency limit of the IMPS spectrum (I_1_/I_2_ in Figure 11a) is related to these constants by the following relation: I_1_/I_2_ = K_ct_/(K_rec_ + K_ct_). The frequency of light intensity modulation, corresponding to the maximum of the semicircle located in the first quadrant (f_max_ in Figure 11a), allows us to find the sum (K_rec_ + K_ct_) from the following relation: 2πf_max_ = (K_rec_ + K_ct_). The values of K_ct_,calculated from the data in Figure 11a for the ZnO(0.07)/Fe_2_O_3_/FTO, ZnO(0.87)/Fe_2_O_3_/FTO, and ZnO(0.2)/Fe_2_O_3_/FTO photoanodesamples in 0.1 M KOH, i.e., during water photoelectrooxidation, are 4.2, 6.5, and 11.5 s^−1^, and the K_rec_ constants are 34.4, 32.1, and 27.1 s^−1^, respectively.

Further, similar IMPS dependences were obtained for the ZnO(0.2)/Fe_2_O_3_/FTO photoanode in 0.1 M KOH solution containing 20% methanol, ethylene glycol or glycerol. These data are illustrated inFigure 11b.

As follows from the figure, in the background electrolyte at the photoanode potential 0.5 V, the photocurrent determined as the point of intersection of the IMPS curve with the *x* axis at low frequencies (LF), is about 30%, and the recombination losses reach 70% of the current of the holes generation evaluated from the intersection of the IMPS curve with the *x* axis at high frequencies (HF). The generation current is the flux of minor photoexcited charge carriers from the bulk of the semiconductor to its surface expressed in electrical units (i.e., the current in the absence of surface hole recombination).The introduction of alcohol into an aqueous solution of 0.1 M KOH reduces the recombination losses at the photoanode from 70 to 50% in the case of CH_3_OH, from 70 to 20% in the case of C_2_H_4_(OH)_2_ and almost to zero in the case of C_3_H_5_(OH)_3_ (Figure 11b, curves *2*–*4*). This reduction is due to the predominant contribution of the photoinduced holes to the process of photoelectrochemical oxidation of alcohol. The values of K_rec_ and K_ct_ calculated from the IMPS data for the water and alcohol photoelectrooxidation are given in Table 2. As seen from the table, the chemical nature of the depolarizer affects both constants. Thus, during the alcohol photoelectrooxidation, a significant increase in K_ct_ is observed, compared to water oxidation. The accelerated consumption of holes entering the photoanode SS in the reaction of alcohol photoelectrooxidation, in turn, is the reason for the decrease in K_rec_. From the voltammetric curves and the IMPS data obtained, it follows that the recombination losses on ZnO-modified hematite photoanodes are higher in the case of methanol and ethylene glycol oxidation as compared to glycerol. It can be assumed that the decrease in the recombination losses during the glycerol photoelectrooxidation is due to its stronger adsorption on the surface of the composite photoanode compared to water, methanol, and ethylene glycol. A similar observation for the same series of alcohols was made when studying the photoelectrocatalytic properties of the nanocrystalline hematite photoanode promoted by titanium [20].

## 3. Materials and Methods

Chemically pure (>99%) ferric chloride FeCl_3_∙6H_2_O, zinc chloride ZnCl_2_, potassium fluoride KF∙2H_2_O, potassium chloride KCl, and hydrogen peroxide H_2_O_2_ (35%) were purchased from Aldrich (St. Louis, MO, USA) and used in the film coating fabrication without further purification. To manufacture the photoanodes, glass substrates with an electrically conductive coating of fluorine-stabilized tin dioxide (F:SnO_2_, FTO) were used (Aldrich; specific resistance ≈ 7 Ω cm^−2^).

### 3.1. Preparation of Hematite and Modified Hematite Films

#### 3.1.1. Preparation of the Photoanodes from Hematite

Polycrystalline α-Fe_2_O_3_ photoanodes were formed on an FTO-coated glass substrate. The substrate was preliminarily cleaned in an ultrasonic bath using the following solvents sequentially: acetone, isopropyl alcohol, and distilled water. The treatment time in each solvent was 15 min. The cleaned substrate was fixed in a Teflon frame in such a way that the surface covered with hematite was 1 cm^2^; the perimeter of the substrate, not intended for coating, was fixed in a titanium current lead. A three-electrode system was assembled on a Teflon cover of the electrochemical cell. A Pt_90_Ir_10_ alloy plate with a geometric area of 8 cm^2^ was used as an anode; a silver chloride electrode served as a reference electrode. The distance between the anode and the cathode was 3 cm. The assembled electrode block was placed in a 100mL thermostated cell equipped with a water jacket. The electrolyte solution (12.5 mM FeCl_3_, 50 mM KF, 0.1 M KCl, and 1 M H_2_O_2_) was poured into the cell immediately before the deposition of α-Fe_2_O_3_. A hematite film was formed for 5 min by electrodeposition at a constant potential E = −0.35 V (vs. Ag/AgCl) and an electrolyte temperature of 70 °C. The amount of electricity passed during the deposition of hematite was about 3 C. During the electrodeposition of oxide films, a PAR 273 (Princeton Applied Research, Oak Ridge, TN, USA) with appropriate software was used as a potentiostat.

The mechanism of hematite film formation by cathodic electrodeposition is described in many works [36,37] and can be represented by the equations:

F^−^ + Fe^3+^ → FeF^2+^ (1)

H_2_O_2_ + 2 e^−^ → 2OH^−^ (2)

FeF^2+^ + 3OH^−^ → FeOOH ↓ + F^−^ + H_2_O (3)

and an overall reaction

3H_2_O_2_ + 2FeF^2+^ + 6e^−^ → 2 FeOOH ↓ + 2F^−^ + 2H_2_O (4)

As a result of the electrodeposition, a yellow FeOOH film was obtained on the glass substrate. The latter was thoroughly washed with distilled water, dried at room temperature, and annealed in air for 2 h in a tube furnace at 500 °C. The temperature was then raised to 750 °C for 10 min. After cooling in the oven for 12 h, the obtained samples with a uniform red α-Fe_2_O_3_ film were used for further investigation. The film thickness was in the range 600–800 nm. 

#### 3.1.2. Modification of Hematite Films

Zinc oxide was deposited on the formed α-Fe_2_O_3_ film at a constant potential E = −1.0 V (vs. Ag/AgCl) from a 5 mM ZnCl_2_ + 0.1 M KCl solution while stirring the electrolyte and saturating it with oxygen. The same cell with a Pt–Ir alloy anode was used. Depending on the deposition time, the amount of electricity passed varied between 50 and 900 mC. After washing and drying, the samples were calcined in an air oven at 400 ° C for 1 h and then cooled as described above. The increase in the thickness of the previously formed hematite film upon deposition of zinc oxide ranged from 2.5 to 32% of the initial thickness, depending on the amount of electricity spent on the ZnO deposition. In particular, the thickness of the zinc oxide layer for the ZnO(0.2)/Fe_2_O_3_/FTO sample is estimated to be 50–60 nm. Zn/Fe weight ratioin hematite samples modified with zinc oxidecalculated on the base of the deposited film thickness is given in Table S1 in the Supplementary Materials section.

The prepared photoanodes with a hematite film modified with zinc oxide were used in further experiments.

Film photoanode samples comprising only zinc oxide (ZnO/FTO) were fabricated by the same electrodeposition procedure; the amount of electricity consumed for the ZnO electrodeposition in this case was 2.0–2.2 C, and the resulting film thickness was about 500–600 nm. 

### 3.2. Characterization of Hematite Films

#### 3.2.1. X-ray Diffraction

The phase composition of the deposited film coatings was studied by X-ray diffraction (XRD) analysis on an Empyrean X-ray diffractometer (Panalytical BV, Almelo, The Netherlands). Ni-filtered Cu-Kα radiation was used; the samples were studied in the Bragg–Brentano geometry. Experimental diffraction patterns were processed using the Highscore program; the phase composition was identified using the ICDD PDF-2 diffraction database. The average size of crystallites of the identified phase was determined from the broadening of the observed diffraction peaks using Williamson–Hall and Scherrer methods.

#### 3.2.2. X-ray Fluorescence 

The spatial distribution of Fe, Zn and other elements on the glass substrate was studied by energy-dispersive X-ray fluorescence (XRF) microscopy. Chemical mapping of Fe and Zn was performed using an XGT-7200V X-ray fluorescence microscope (Horiba, Kyoto, Japan) equipped with a Rh tube operating at 1 mA and 15 kV accelerating voltage. A 1.2 mm monocapillary was used to confine the beam. To improve the map quality strong overlap of the measurement spots was applied.

#### 3.2.3. Absorption Spectra

Absorption spectra of the obtained films were studied in a range of 300–700 nm at room temperature using a Lambda35 Perkin Elmer spectrometer (Renishaw, New Mills/Wotton-under-Edge UK). 

#### 3.2.4. Raman Spectra

Raman spectra were recorded using an inVia “Reflex” Raman spectrometer (Renishaw, New Mills/Wotton-under-Edge, UK) with a 50× objective. The 405 nm line of a diode laser was used for excitation, and the laser power on the sample was less than 0.2 mW. Microphotographs were taken using a Leica microscope with a 50× objective, on the basis of which the Raman system was assembled. 

#### 3.2.5. Film Thickness Measurement

The thickness of the electrodeposited films was determined on a Thin Films measurement system MProbe 20 (Semiconsoft Inc., Southborough, MA, USA). The measurement range was from 1 nm to 1 mm.

#### 3.2.6. Photoelectrochemical measurements

Photoelectrochemical measurements were performed using a setup comprising photoelectrochemical three-electrode cell PECC-2 (Zahner Elektrik, Kronach, Germany), a 150 W solarspectrum simulator 96000 (Newport, Irvine, CA, USA) with an AM1.5G filter, and an IPC-Pro MF potentiostat (IPChE RAS, Moscow, Russia). The working electrode in the cell was a 1 cm^2^ photoanode with a film coating made of hematite or hematite doped with zinc oxide. A Pt wire with a surface area of ≈3 cm^2^ was used as an auxiliary electrode. A silver chloride electrode was used as a reference, relative to which all potentials are given in this work. Potentials relative to a reversible hydrogen electrode can be determined from the equation: E_RHE_ = E_Ag/AgCl_ + 0.059 × pH + E°_Ag/AgCl_, where E°_Ag/AgCl_ = 0.197 [13]. Illumination was directed from the photoanode backside. The illumination power density at different distances from the light source was determined using a Nova instrument (OPHIR-SPIRICON Inc., Jerusalem, Israel). Photoelectrochemical oxidation of organic substrates on the prepared photoanodes was carried out under visible light illumination of 1 sun at a power density of 100 mW cm^−2^. 

Intensity-modulated photocurrent spectroscopy (IMPS) data were obtained using a Zahner CIMPS computerized photo-electrochemical workstation (Zahner-Elektrik Gmbh & Co.KG, Kronach, Germany). The station was equipped with a TLS03 monochromatic light source with a set of LEDs with wavelengths from 320 to 1020 nm and the CIMPS-QE/IPCE software package. Incident photon-to-current conversion efficiency (IPCE) data were collected in the wavelength range 350−800 nm with 10 nmspectral resolution. The IMPS spectra were recorded under the illumination of the photoanode with monochromatic light with a wavelength of 452 nm and a fixed intensity of 14 mW cm^−2^ in the frequency range from 0.02 to 2 × 10^3^ Hz. A sinusoidal disturbance (~10% of stationary illumination) was superimposed on a constant base light intensity. Normalized IMPS curves were obtained by dividing the real Re(I_ph_) and imaginary Im(I_ph_) components of the experimental IMPS curve by the value of I_2_,where I_2_ corresponds to the Re(I_ph_) maximum value.

## 4. Conclusions

A ZnO/Fe_2_O_3_ composite thin film was prepared by electrochemical deposition on a conductive glass substrate. It is shown that modification of the hematite film with zinc oxide leads to a significant improvement in the electrocatalytic properties of the photoanode in the reaction of water oxidation. Depending on the ZnO content, the increase in photoelectrooxidation current density at a photoanode potential of 1.23 V (vs. RHE) can reach an order of magnitude compared to the photoanode with a pure hematite film. The obtained results can be explained by an increase in the density of surface states of a hematite photoelectrode upon its modification with zinc oxide. The ZnO additive contributes both to a decrease in the recombination losses and to an increase in the efficiency of charge transfer to depolarizer (water) molecules. The maximum improvement in the photoelectrocatalytic properties of the electrode is achieved with anamount of a modifying additive (ZnO) corresponding to 0.2 C cm^−2^ of electricity spent during its electrodeposition. It was also found that a nanocomposite film of ZnO-modified hematite accelerates the degradation of structurally different alcohols. This effect may be explained by more efficient charge transfer to the alcohol molecules than to the H_2_O ones. The effect is the strongest in the case of the photoelectrooxidation of glycerol under visible light illumination. The rate constant of the charge transfer in this reaction is much higher than the values of constants for water oxidation and the photogenerated charge carriers recombination. The increase in the photoelectrooxidation rate in the sequence H_2_O < CH_3_OH < C_2_H_4_(OH)_2_ < C_3_H_5_(OH)_3_ can be explained by the influence of the depolarizer nature on both the photoelectrooxidation rate constant (transfer of holes to the depolarizer) and the recombination processes on the surface states of the hematite photoanode modified with zinc oxide.

## Figures and Tables

**Table 1:** Estimation of the mole % of Zn in samples modified with zinc oxide, where Q_dep_ is the amount of electricity spent on electrodeposition; L is the average thickness of the oxide layer; P is the weight of metal in the oxide; M is the amount of metal in moles; and [Zn]/([Zn] + [Fe]) is mol% of Zn in the sample. The geometric surface area of the photoanode is 1 cm^2^; specific gravity is 5.1 and 5.6 g cm^−3^ for Fe_2_O_3_ and ZnO, respectively.

Sample	Q_dep_,C cm^−2^	L, cm	P, g	M	[Zn]/([Zn] + [Fe]) mol %
Fe_2_O_3_/FTO	3 *	7 × 10^−5^ *	2.5 × 10^−4^ *	44.8 × 10^−7^	
ZnO(0.07)/Fe_2_O_3_/FTO	0.07 **	1.75 × 10^−6^ **	7.8 × 10^−6^ **	1.2 × 10^−7^	2.6
ZnO(0.2)/Fe_2_O_3_/FTO	0.2 **	5.5 × 10^−6^ **	2.5 × 10^−5^ **	4.6 × 10^−7^	9.3
ZnO(0.87)/Fe_2_O_3_/FTO	0.87 **	2.24 × 10^−5^ **	10^−4^ **	15 × 10^−7^	25

* The data refer to the α-Fe_2_O_3_ layer electrodeposited onto FTO glass for all samples; ** The data refer to the ZnO layer electrodeposited onto the α-Fe_2_O_3_ layer.

**Table 2:** Charge transfer (K_ct_) and recombination (K_rec_) rate constants for the ZnO(0.2)/Fe_2_O_3_/FTO film photoanode at E = 0.5 V (vs. Ag/AgCl) under monochromatic light (452 nm) illumination at a 14 mW cm^−2^ power density in aqueous solutions of 0.1 M KOH, 0.1 MKOH + 20%CH_3_OH, 0.1 M KOH + 20% C_2_H_4_(OH)_2_, and 0.1 M KOH + 20% C_3_H_5_(OH)_3_.

Depolarizer	K_ct_, s^−1^	K_rec_, s^−1^
H_2_O	11.5	27.1
CH_3_OH	21.6	17
C_2_H_4_(OH)_2_	41.1	11.6
C_3_H_5_(OH)_3_	K_ct_ >> K_rec_	-

## Data Availability

All results were generated during this study.

## Supplementary Materials

The supporting information can be downloaded at: https://www.mdpi.com/article/10.3390/ijms241814046/s1.
