# Supplementary material for: Photoelectrocatalytic Activity of ZnO-Modified Hematite Films in the Reaction of Alcohol Degradation
Source: Int J Mol Sci. 2023 Sep 13;24(18):14046. doi: 10.3390/ijms241814046 (PMC10531269; doi:10.3390/ijms241814046)
Supplement: Supplementary file 1 [file ijms-24-14046-s001.zip › ijms-2567692-supplementary.pdf]

Photoelectrocatalytic Activity of ZnO-Modified Hematite Films in the Reaction of Alcohol Degradation

Vitali A. Grinberg * , Victoor V. Emets , Natalia A. Mayorova, Aleksey A. Averi in and Andrei A. Shiryaev

Frumkin Institute of Physical Chhemistry and Electrochemistry, Russian Academy of SScciences, Leninsky Prospekt 31, Building 4, 119071 Moscoww, Russia

* Correspondence: vgrinberg@phyche.ac.ru; vitgreen@mail.ru

Supplementary materials

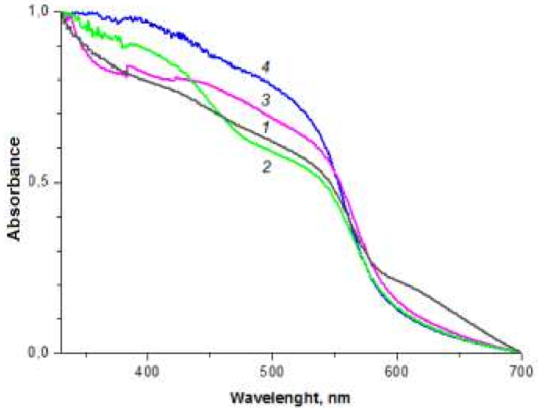

Figure S1. Normalized absorption spectra for the film photoanodes: (*1*) Fe_2O_3/FTO; (*2*) ZnO(0.07)/Fe_2O_3/FTO; (3) ZnO(0.2)/Fe_2O_3/FTO; and (4) ZnO(0.87)/Fe_2O_3/FTO.

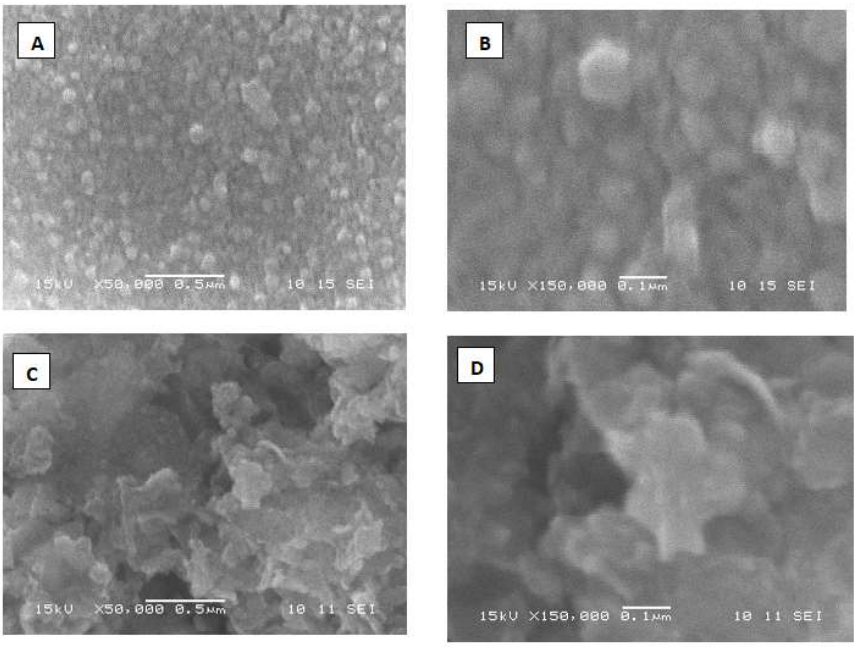

Figure S2. SEM images of the hematite films modified with different amounts of electrodeposited ZnO: (A,B) ZnO(0.2)/Fe_2O_3/FTO; and (C,D) ZnO(0.87)/Fe_2O_3/FTO. Conditions of the samples preparation are described in section 3.1.

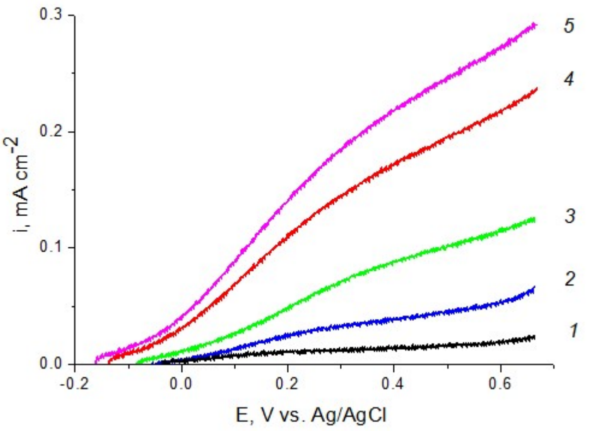

Figure S3. Voltammograms of the ZnO(0.07)/Fe_2O_3/FTO film photoanode obtained: (*1*) in "dark" conditions, and (*2* - *5*) under visible light illumination with a power density of 100 mW cm^{-2} in aqueous solutions of (*2*) 0.1 M KOH; (*3*) 0.1 M KOH + 20% CH_3OH; (*4*) 0.1 M KOH + 20% $C_2H_4(OH)_2$; and (*5*) 0.1 M KOH + 20% $C_3H_5(OH)_3$. The dark curves for all solutions practically coincide. Potential scan rate is 10 mV s^{-1}.

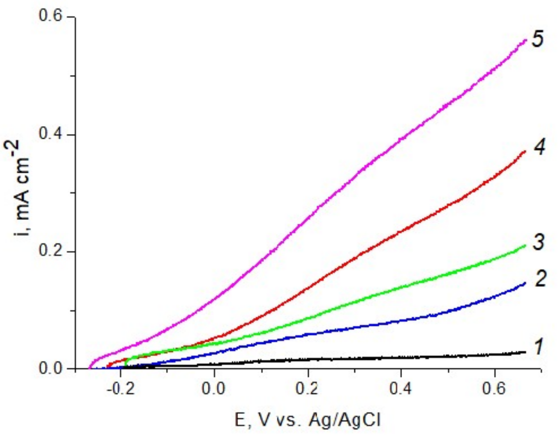

Figure S4. Voltammograms of the ZnO(0.87)/Fe_2O_3/FTO film photoanode obtained: (*1*) in "dark" conditions, and (*2* - *5*) under visible light illumination with a power density of 100 mW cm^{-2} in aqueous solutions of (*2*) 0.1 M KOH; (*3*) 0.1 M KOH + 20% CH_3OH; (*4*) 0.1 M KOH + 20% $C_2H_4(OH)_2$; and (*5*) 0.1 M KOH + 20% $C_3H_5(OH)_3$. The dark curves for all solutions practically coincide. Potential scan rate is 10 mV s^{-1}.

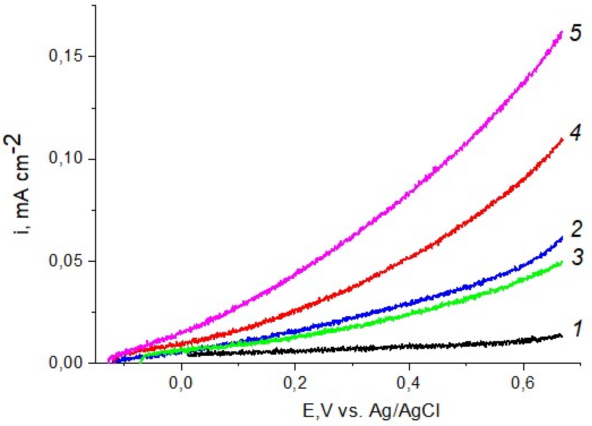

Figure S5. Voltammograms of the Fe_2O_3/FTO film photoanode obtained: (*1*) in "dark" conditions, and (*2* - *5*) under visible light illumination with a power density of 100 mW cm^{-2} in aqueous solutions of 0.1 M KOH (*2*); 0.1 M KOH + 20% CH_3OH (*3*); 0.1 M KOH + 20% $C_2H_4(OH)_2$ (*4*); and 0.1 M KOH + 20% $C_3H_5(OH)_3$ (*5*). The dark curves for all solutions practically coincide. Potential scan rate is 10 mV s^{-1}.

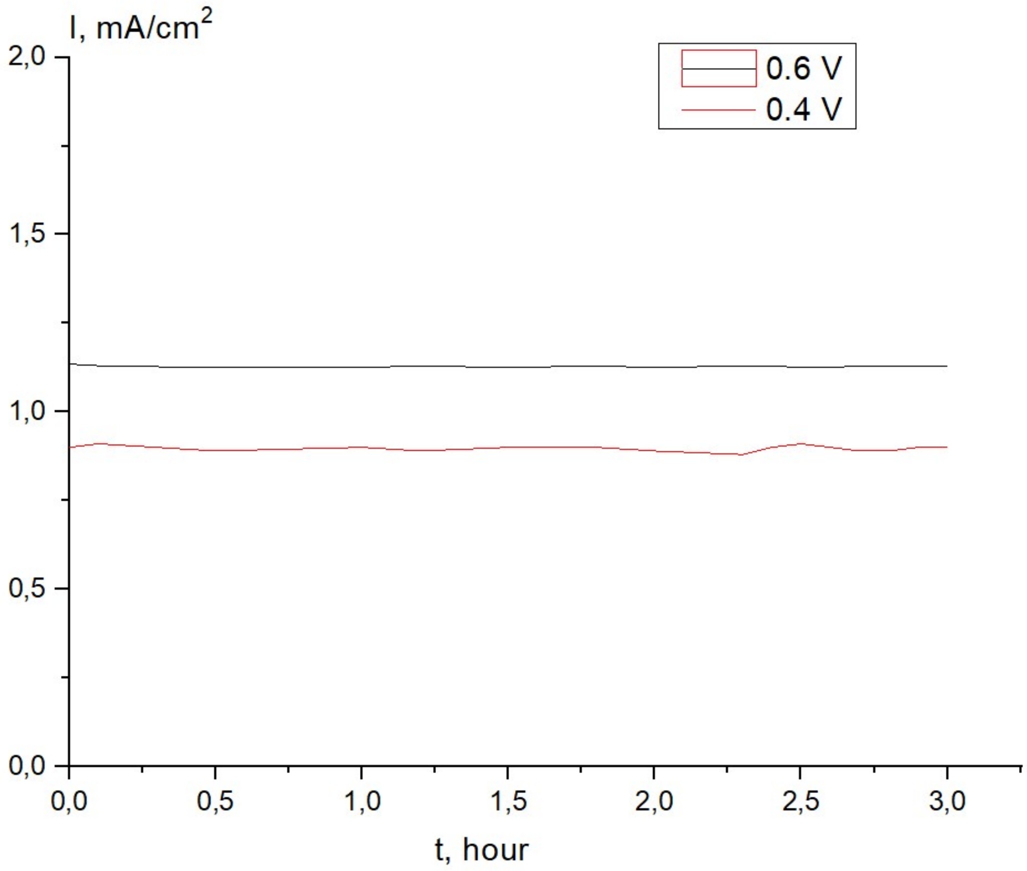

Figure S6. Chronoamperograms obtained at the ZnO(0.2)/Fe_2O_3/FTO film photoanode at the bias potentials 0.4 V and 0.6 V vs. Ag/AgCl in 0.1 M KOH + 20% $C_3H_5(OH)_3$ aqueous solution under visible light illumination with a power density of 100 mW cm^{-2} .

Table S1. Calculation of the Zn / Fe weight ratio in hematite samples modified with zinc oxide, where: Q_{dep} is the amount of electricity spent on the oxide deposition; L is an average thickness of the oxide layer; d is specific gravity of the oxide; S is the geometric surface area of the photoanode sample; P is the weight of the metal in the oxide; and $WR_{Zn:Fe}$ is the Zn:Fe weight ratio in the sample.

Sample	Q_{dep}, C cm^{-2}	L, cm	d, g cm^{-3}	S, cm^{-2}	P, g	$WR_{Zn:Fe}$
Fe_2O_3/FTO	3*	7×10^{-5} *	5.1*	1	2.5×10^{-4} *	
ZnO(0.07)/Fe_2O_3/FTO	0.07**	1.75×10^{-6}**	5.6**	1	7.8×10^{-6} **	3.12×10^{-2}
ZnO(0.2)/Fe_2O_3/FTO	0.2**	5.5×10^{-6} **	5.6**	1	2.5×10^{-5} **	10^{-1}
ZnO(0.87)/Fe_2O_3/FTO	0.87**	2.24×10^{-5}**	5.6**	1	10^{-4} **	4×10^{-1}

* The data refer to the α-Fe_2O_3 layer electrodeposited onto FTO glass for all samples;

** The data refer to the ZnO layer electrodeposited onto the α-Fe_2O_3 layer.
